# Advances in the Management of Pancreatic Cancer: Current Strategies and Emerging Therapies

**DOI:** 10.3390/ijms26157055

**Published:** 2025-07-22

**Authors:** Supriya Peshin, Ehab Takrori, Naga Anvesh Kodali, Faizan Bashir, Sakshi Singal

**Affiliations:** 1 Department of Internal Medicine, Norton Community Hospital, Norton, VA 24273, USA; 2 College of Medicine, Alfaisal University, Riyadh 11533, Saudi Arabia; etakrori@gmail.com; 3 Department of Surgery, Wake Forest School of Medicine, Winston-Salem, NC 27157, USA; anveshkodali@gmail.com; 4 School of Medicine, Shiraz University of Medical Sciences, Shiraz 71348-45794, Iran; faizanbashir.med@gmail.com; 5 Department of Hematology & Oncology, East Tennessee State University, Johnson City, TN 37614, USA

**Keywords:** pancreatic ductal adenocarcinoma, chemotherapy, targeted therapy, immunotherapy, mRNA vaccine, surgical resection, neoadjuvant therapy

## Abstract

Pancreatic ductal adenocarcinoma (PDAC) remains a formidable malignancy with rising incidence and dismal long-term survival, largely due to late-stage presentation and intrinsic resistance to therapy. Recent advances in the multidisciplinary management of PDAC have reshaped treatment paradigms across disease stages. For localized disease, innovations in surgical techniques and the adoption of neoadjuvant strategies have improved resection rates and survival outcomes. In metastatic settings, multiagent chemotherapy regimens and precision therapies targeting BRCA mutations and rare gene fusions are expanding treatment options. Immunotherapeutic modalities, including checkpoint inhibitors, adoptive cell therapies, and mRNA vaccines, show emerging promise despite PDAC’s traditionally immunosuppressive microenvironment. This review synthesizes the current evidence on established therapies and critically evaluates novel and investigational approaches poised to redefine the therapeutic landscape of pancreatic cancer.

## 1. Introduction

Pancreatic cancer remains one of the deadliest malignancies globally, with pancreatic ductal adenocarcinoma (PDAC) constituting the predominant histological subtype [1,2]. Characterized by rapid progression and early dissemination, PDAC is typically detected in its late stages [1,3]. In 2024, the U.S. reported 66,440 new cases and 51,750 deaths, accounting for 8.6% of cancer-related mortality. Despite ongoing efforts, the five-year survival rate from 2014 to 2020 remains low at 12.8%. At diagnosis, only 15–20% of patients have resectable tumors, while 25–30% present with locally advanced disease and 50–60% already exhibit distant metastasis [4]. The pancreas’s retroperitoneal location facilitates early vascular invasion, limiting surgical options and therapeutic efficacy. Additionally, molecular heterogeneity and a dense, immunosuppressive stroma further impede treatment response [5,6].

The development of PDAC is driven by a sequence of genetic alterations and progressive histopathological changes. Mutations in KRAS are the most common and occur early in pancreatic tumorigenesis, followed by the inactivation of tumor suppressor genes such as CDKN2A, TP53, and SMAD4 [5,6]. These driver gene mutations contribute to uncontrolled cell proliferation, resistance to apoptosis, the evasion of immune surveillance, and desmoplastic stroma formation. An understanding of these alterations has informed diagnostic biomarker development and created new therapeutic opportunities, particularly in precision oncology [6,7,8].

Advancements in imaging and surgical techniques have refined PDAC classification into resectable (R), borderline (BR), locally advanced, and metastatic (Table 1) [9,10,11]. Given the prognostic importance of the margin status and the surgeon’s expertise, neoadjuvant therapy is increasingly favored for borderline and high-risk cases [12]. While surgical resection remains the primary curative approach, only ~20% of patients are eligible at diagnosis [11,13]. Minimally invasive and robotic-assisted approaches have improved perioperative outcomes [14,15], while ablative therapies are emerging as alternatives for inoperable cases [14,16,17,18]. Chemotherapy and radiotherapy remain essential for non-surgical management [19,20,21,22]. This review synthesizes the current treatment strategies and critically evaluates novel and investigational therapies that may improve survival and quality of life for patients with pancreatic cancer.

## 2. Current Treatment Options

### 2.1. Localized Disease

Pancreatic cancer remains a leading cause of cancer-related mortality in the United States, with most cases diagnosed at an advanced or unresectable stage [23]. However, approximately 15–20% of patients present with localized disease potentially eligible for curative interventions [4]. In these cases, a multidisciplinary approach involving surgical, medical, and radio-oncological expertise has been shown to improve treatment outcomes and survival [24].

### 2.2. Neoadjuvant Chemotherapy

Neoadjuvant chemotherapy has become increasingly common for borderline resectable pancreatic cancer (BRPC) and locally advanced pancreatic cancer (LAPC), with studies showing improved outcomes [25]. Several clinical studies have demonstrated that initiating treatment with neoadjuvant chemotherapy can improve outcomes in these patient populations. Following treatment, patients are reassessed for surgical eligibility based on imaging findings and the clinical status. In the absence of disease progression or metastasis, resection may be performed [26]. Notably, limited radiographic tumor shrinkage does not preclude surgical candidacy, as imaging alone may not fully reflect the treatment response. This is particularly evident in patients receiving intensive neoadjuvant regimens such as FOLFIRINOX—a multiagent chemotherapy comprising of folinic acid (leucovorin), fluorouracil (5-FU), irinotecan, and oxaliplatin. Prospective clinical studies, including a major trial discussed below, have shown that even when tumors exhibit minimal radiographic shrinkage, patients may still achieve significant histopathologic regression and undergo successful R0 resection. These findings highlight the importance of clinical judgement and multidisciplinary reassessment, rather than relying solely on imaging when evaluating resectability after neoadjuvant therapy.

The NUPAT-01 trial demonstrated favorable outcomes in BRCP patients using two chemotherapy regimens—FOLFIRINOX and gemcitabine plus nab-paclitaxel (GEM/nab-PTX)—achieving R0 resection in 67.4% of patients and a median overall survival (mOS) period of 39.4 months. Similarly, a trial by Jang et al. showed that preoperative chemotherapy significantly increased the likelihood of margin-negative resections [25].

Real-world data from the National Cancer Database indicate a survival benefit with neoadjuvant therapy in early-stage (Stage I/II) pancreatic cancer, showing mOS of 26 months vs. 21 months with immediate surgery (hazard ratio = 0.72, *p* < 0.01) [26]. However, a meta-analysis of six RCTs comprising 805 patients with resectable pancreatic cancer found no significant improvement in disease-free or overall survival with neoadjuvant gemcitabine-based chemotherapy or chemoradiation compared to upfront surgery followed by adjuvant therapy. These conflicting findings highlight ongoing uncertainty around optimal treatment sequencing and the need for improved patient selection [27].

Neoadjuvant therapy offers several theoretical and practical advantages in pancreatic cancer management. Given that up to 40–50% of patients may not complete planned adjuvant chemotherapy due to postoperative complications or functional decline, initiating systemic therapy preoperatively ensures broader access to multimodal treatment [23]. Biologically, neoadjuvant treatment targets micrometastatic disease early and provides insights into tumor responsiveness, potentially avoiding unnecessary surgery in non-responders. However, its role in clearly resectable cases remains under investigation, with concerns about disease progression during the neoadjuvant period in poor responders [27].

### 2.3. Surgical Options

The National Comprehensive Cancer Network (NCCN) classifies PDAC into three categories based on resectability: resectable, borderline resectable, and locally advanced, based on vascular involvement. Resectable tumors have no or ≤180° contact with critical vessels such as the superior mesenteric vein (SMV), portal vein (PV), or major arteries without contour irregularity. Borderline resectable tumors involve limited vascular contact, potentially allowing for reconstruction. Locally advanced tumors exhibit extensive vascular encasement and are not candidates for upfront surgery, although select cases may be downstaged with neoadjuvant therapy. Metastatic disease is considered separately under clinical staging and is generally managed with systemic therapy rather than surgical intervention [28].

The surgical approach is largely dictated by the tumor location: distal pancreatectomy is used for tumors of the body and tail, while pancreaticoduodenectomy (PD) is performed for tumors in the head or uncinate process [29]. Advances in vascular resection, minimally invasive, and robotic-assisted techniques have broadened the surgical armamentarium and improved recovery in selected patients, including reduced blood loss and shorter hospital stays [15]. However, recurrence remains a substantial challenge, often due to micrometastatic disease present at diagnosis [30].

Notably, the MD Anderson classification is more inclusive than the NCCN framework, categorizing some tumors with limited vascular involvement as resectable if reconstruction is feasible and expanding the borderline resectable category to include tumors with adjacent organ involvement, thereby broadening surgical eligibility [28]. In addition to the anatomical definitions provided by the NCCN, the 2017 International Consensus Criteria for borderline resectable PDAC recommend incorporating biological and conditional factors to improve treatment stratification [10]. Biological factors include elevated serum CA 19-9 levels that may suggest micrometastatic disease, while conditional factors include the patient performance status and comorbidities that may impact surgical candidacy. This multidimensional approach complements anatomic staging and supports the tailored use of neoadjuvant therapy.

### 2.4. Adjuvant Chemotherapy

Despite successful surgical resection, long-term outcomes in PDAC remain poor. Surgery alone yields a 5-year survival rate of approximately 20%, which improves to 30–40% with the addition of adjuvant chemotherapy [31,32]. Over the past two decades, robust clinical trial data have firmly established adjuvant systemic therapy as a standard component of curative-intent treatment for resected, non-metastatic PDAC [33].

Adjuvant therapy selection is primarily based on the patient’s performance status. Patients with good functional capacity (ECOG 0–1) are treated with modified FOLFIRINOX—a multiagent regimen including 5-fluorouracil (5-FU), oxaliplatin, irinotecan, and leucovorin—due to its superior survival benefits. Those with lower performance may receive single-agent chemotherapy, such as gemcitabine or a fluoropyrimidine, or gemcitabine in combination with capecitabine [28].

Gemcitabine’s role in adjuvant therapy was established by the *CONKO-001 trial*, which showed improved mOS to 22.8 months vs. 20.2 months with observation following surgery (HR 0.76; 95% CI, 0.61–0.95; *p* = 0.01) [34,35]. The earlier *ESPAC-1 trial* demonstrated a survival benefit with 5-FU/leucovorin, increasing the mOS from 14.0 to 19.7 months (HR 0.66; 95% CI, 0.52–0.83; *p* = 0.0005) [35]. Building on this, the *ESPAC-4 phase III* trial reported that gemcitabine combined with capecitabine modestly outperformed gemcitabine alone, significantly improving mOS to 28.0 months compared to 25.5 months with gemcitabine alone (HR 0.82; 95% CI, 0.68–0.98; *p* = 0.032), establishing it as the preferred adjuvant regimen [35].

JASPAC 01 was a breakthrough phase III randomized controlled trial conducted in Japan to compare adjuvant S-1 vs. gemcitabine in patients with resected pancreatic cancer. The results showed a significant improvement in overall survival with S-1; after 5 years, the S-1 group had 44.1% overall survival, compared to 24.4% in the gemcitabine group (HR 0.57; *p* < 0.0001). JASPAC 01’s findings confirmed S-1’s superior efficacy as an adjuvant chemotherapy option in East Asian populations [36].

Similarly, gemcitabine combined with cisplatin showed encouraging results in a small phase II study of 22 patients, achieving an mOS period of 35.5 months. Nonetheless, this came at the cost of significant toxicity, with grade 3/4 adverse events reported in nearly 59% of patients, limiting its broader applicability [37].

The *APACT trial* evaluated gemcitabine plus nab-paclitaxel vs. gemcitabine monotherapy in the adjuvant setting. While the primary analysis did not demonstrate a statistically significant difference, a subsequent 2021 update revealed improved survival in the combination arm (mOS 41.8 vs. 37.7 months; *p* = 0.0091) [38].

Among the most compelling evidence in recent years is that from the *PRODIGE-24 trial*, which evaluated modified FOLFIRINOX in patients with an excellent performance status. This regimen significantly improved mOS to 54.4 months compared to 35 months with gemcitabine monotherapy (HR, 0.64; 95% CI, 0.48–0.86; *p* < 0.001), establishing it as the current standard for fit patients [32,39].

#### 2.4.1. Radiotherapy and Neoadjuvant Strategies

In addition to adjuvant chemotherapy, interest has grown in neoadjuvant and perioperative strategies, including radiotherapy, particularly for borderline resectable disease. The role of radiation therapy in the management of PDAC remains a subject of ongoing debate, with its optimal use still under clinical investigation. Neoadjuvant chemoradiation has demonstrated potential benefits in certain settings, particularly in resectable and borderline resectable disease.

Table 2 summarizes key neoadjuvant and perioperative trials that have shaped the current management approaches, including both chemotherapy-only and chemoradiation strategies, across resectable and borderline resectable populations.

The *PREOPANC trial* demonstrated improved overall survival with gemcitabine-based neoadjuvant chemoradiotherapy compared to upfront surgery, benefiting both resectable and borderline resectable PDAC [31,40]. Similarly, the *ESPAC-5F* phase II trial reported higher one-year survival with chemoradiation, supporting its use in selected patients.

Retrospective data have echoed these findings, suggesting improved R0 resection rates and survival outcomes with neoadjuvant chemoradiation. However, more recent prospective evidence has cast doubt on its routine use. The *ALLIANCE A021501 trial* compared modified FOLFIRINOX alone vs. mFOLFIRINOX followed by hypofractionated radiotherapy in borderline resectable PDAC. The addition of radiation did not confer a survival advantage, underscoring the need for careful patient selection [41].

Additional evidence supporting neoadjuvant strategies comes from a retrospective multicenter study by Macedo et al. evaluated neoadjuvant FOLFIRINOX vs. gemcitabine/nab-paclitaxel in borderline resectable and locally advanced pancreatic cancer. The study found that patients achieving a ≥ 50% decline in CA 19-9 experienced significantly improved overall survival, with a median OS period of 42.3 months. The pathologic response correlated with survival outcomes, emphasizing the prognostic value of biomarker-driven response assessment in neoadjuvant therapy [42].

Similarly, the NEONAX trial explored perioperative vs. adjuvant gemcitabine plus nab-paclitaxel in patients with resectable PDAC. Although the primary endpoint was not met, the trial reported longer median disease-free survival (DFS) and overall survival in the perioperative group, supporting the further investigation of neoadjuvant strategies in this setting [43].

The *ESPAC-1 trial* raised concerns about adjuvant radiation, showing worse outcomes with combined 5-FU-based chemotherapy in the postoperative setting. Likewise, the *LAP-07 trial* in locally advanced, unresectable PDAC found no survival benefit from adding capecitabine-based chemoradiation after gemcitabine therapy, despite a modest improvement in median progression-free survival (PFS), suggesting a limited role for chemoradiation in this context [44].

Chemoradiation may be selectively used after an R1 resection or as part of a neoadjuvant therapy in borderline resectable or locally advanced PDAC, ideally within a multidisciplinary team. While smaller studies suggest that it may improve R0 resection rates, this has not been confirmed in larger trials. Notably, the *SWOG trial* showed no added benefit from combining radiation with FOLFIRINOX, and its use in the neoadjuvant setting is not currently recommended [45].

Carbon ion radiotherapy (CIRT) has emerged as a novel treatment paradigm for patients with locally advanced unresectable pancreatic cancer (URPC), particularly for patients unresponsive to or resistant to conventional X-ray radiotherapy. A prospective study conducted by Okamoto et al. looked at CIRT alone and in combination with chemotherapy in a cohort of 44 URPC patients, most of whom received prior neoadjuvant chemotherapy. The results were encouraging, with a median overall survival period of 29.6 months with CIRT and 34.5 months from the initiation of initial chemotherapy. The two-year overall survival, local control, and progression-free survival rates were 56.6%, 76.1%, and 29.0%, respectively. These early data suggest that CIRT can provide a significant survivorship benefit, even among patients receiving multiagent chemotherapy [46].

Likewise, in a multicenter retrospective study conducted in Japan, Kawashiro et al. reviewed 72 patients who were treated with CIRT for locally advanced pancreatic cancer and reported a two-year overall survival rate of 46% and median survival of 21.5 months, with a cumulative two-year local recurrence rate of 24% [47]. Another study by Liermann et al. reported high local control rates of 89% at one year following CIRT with or without concurrent gemcitabine in 21 patients, but did not demonstrate a clear improvement in overall survival (median = 11.9 months) [48]. The authors attributed the lack of better overall survival results to the aggressive nature of the disease, particularly in patients who progressed after prior chemotherapy and developed early distant metastases. The authors suggested that combination strategies may need to incorporate systemic therapy. Taken together, these findings support the potential of carbon ion therapy as a valuable local treatment option for LAPC, particularly when integrated into multimodal treatment strategies.

**Table 2:** Key trials of neoadjuvant and perioperative therapies in pancreatic cancer.

Study	Design/n	Treatment Approach (with Neo/Peri Indicated)	Disease Setting	Outcomes
**PREOPANC (Original)** [40]	Phase III/n = 248 (ITT 246)	Neoadjuvant gemcitabine-based chemoradiotherapy vs. upfront surgery + adjuvant gemcitabine	Resectable + borderline resectable	**Median OS:** 15.7 mo. (neo) vs. 14.3 mo (surgery); HR 0.73 (95% CI 0.56–0.96), *p* = 0.025 **5-year OS** 20.5% vs. 6.5%
**NEONAX Trial** [43]	Phase II/n = 127	Perioperative (2 pre-op + 4 post-op cycles) vs. adjuvant (6 cycles) gemcitabine + nab-paclitaxel	Resectable	**Median OS (ITT):** 25.5 mo (periop) (95% CI 19.7–29.7) vs. 16.7 mo (adjuvant) (95% CI 11.6–22.2) **Median DFS:** 11.5 mo (periop) (95% CI 8.8–14.5) vs. 5.9 mo (adjuvant) (95% CI 3.6–11.5) **18-mo DFS:** 33.3% periop vs. 41.4% adjuvant Primary endpoint not met; no HR or *p* reported
**Macedo et al.** [42]	Retrospective multicenter/n = 274	Neoadjuvant FOLFIRINOX vs. gemcitabine + nab-paclitaxel	Borderline resectable + locally advanced followed by resection	**Median OS:** 42.3 mo (≥50% CA 19-9 response) vs. 24.3 mo (<50% CA 19-9) **By pathology**: NR (pCR) vs. 40.3 mo (pPR) vs. 26.1 mo (pLR); *p* < 0.001 **L-RFS:** 27.3 vs. 14.1 mo, *p* = 0.042 **MFS:** 29.3 vs. 13 mo, *p* = 0.047 No significant OS difference between FOLFIRINOX and Gem/NabP

#### 2.4.2. Unresectable or Metastatic Pancreatic Cancer

The management of unresectable or metastatic PDAC requires a multidisciplinary approach tailored to the disease extent, symptoms, and patient performance status. Advanced PDAC follows either local extension and systemic dissemination, both contributing to morbidity and mortality. Notably, up to one-third of patients die from local disease progression without evidence of distant metastasis [7]. Systemic therapy remains the primary treatment, supported by locoregional interventions for palliation and quality of life enhancements [7].

#### 2.4.3. Palliative Surgical and Endoscopic Interventions

In addition to conventional ERCP and surgical bypass, endoscopic ultrasound (EUS)-guided interventions are increasingly utilized in patients with advanced PDAC requiring palliative decompression. EUS-guided biliary drainage (EUS-BD), including hepaticogastrostomy or choledochoduodenostomy, has emerged as a viable alternative when ERCP fails or is anatomically challenging. EUS-BD is associated with high technical success and favorable safety profiles in expert centers and may be particularly beneficial in cases of altered surgical anatomy or duodenal obstruction [49]. Likewise, EUS-guided gastrojejunostomy (EUS-GJ) provides a minimally invasive option for patients with malignant gastric outlet obstruction, offering comparable symptom relief to surgical bypass with a shorter recovery time and promising long-term outcomes [50].

Stent choice is based on prognosis: plastic stents are suitable for patients with a life expectancy < 6 months, while self-expanding metal stents (SEMS) offer longer patency in patients with a better prognosis [50]. For duodenal obstruction, endoscopic stenting provides rapid symptom relief in frail patients, whereas surgical gastrojejunostomy is preferred in patients with a good performance status or concurrent biliary and duodenal obstruction [45].

Pain management is critical in advanced PDAC. For patients with refractory pain, endoscopic or percutaneous celiac plexus neurolysis and thoracoscopic splanchnicectomy are effective options to reduce the opioid burden. These interventions should be tailored based on symptom severity, prognosis, and patient preferences [45,51].

EUS-guided locoregional therapies are also gaining traction in the palliative and investigational treatment of PDAC. EUS-guided fine-needle injection (FNI) enables the local delivery of therapeutic agents—such as chemotherapy, gene therapy vectors, or immunomodulators—directly into the tumor. This approach aims to increase local efficacy while minimizing systemic toxicity. A recent review by Prete and Gonda summarized the current landscape of EUS-guided ablative therapies, particularly radiofrequency ablation and ethanol injection, for pancreatic neuroendocrine tumors and cystic lesions, providing insights into procedural efficacy and safety [52].

Additionally, EUS-guided radiofrequency ablation (EUS-RFA) has emerged as a minimally invasive technique for the ablation of pancreatic tumors. EUS-RFA uses a specialized electrode probe to deliver thermal energy under real-time ultrasound guidance, leading to localized tumor necrosis. A recent review by Khoury et al. outlined the expanding role of EUS-RFA in the management of pancreatic cancer and its integration into multimodal strategies [53]. Early-phase studies suggest that EUS-RFA may improve local control and potentially synergize with systemic treatments. These approaches are currently under clinical evaluation, particularly in patients who are not surgical candidates or as adjuncts to palliative care.

#### 2.4.4. Systemic Chemotherapy in Metastatic PDAC

Systemic therapy is the cornerstone for metastatic PDAC treatment, with multiagent regimens outperforming monotherapy. Regimens such as FOLFIRINOX and gemcitabine-based combinations have demonstrated survival benefits but differ in toxicity and patient eligibility. Table 3 summarizes the comparative efficacy, toxicity, and indications of commonly used chemotherapy protocols in metastatic PDAC (Table 3). FOLFIRINOX offers the greatest survival benefit, extending the median overall survival period to 11.1 months vs. 6.8 months with gemcitabine monotherapy [54].

The MPACT trial established gemcitabine plus nab-paclitaxel as an effective alternative, particularly in high-burden disease, with mOS of 8.5 months and reported long-term survival in select patients [55].

Other gemcitabine-based combinations—including those with 5-FU, erlotinib, cisplatin, or capecitabine—have shown limited or inconsistent survival benefits [56,57,58,59]. The FIRGEM regimen, combining fixed-dose gemcitabine with irinotecan and FOLFIRI.3, improved PFS to 6 months vs. 3.4 months with gemcitabine alone, but was associated with increased hematologic toxicity [60].

The NAPOLI-3 trial established Nalirifox (liposomal irinotecan, 5-FU, leucovorin, oxaliplatin) as a frontline option, showing an improvement over gemcitabine/nab-paclitaxel (11.1 vs. 9.2 months; HR = 0.83; *p* = 0.04), with more gastrointestinal side effects but fewer hematologic events [61]. A meta-analysis of over 2500 patients across seven phase III trials confirmed the longer PFS with Nalirifox and FOLFIRINOX (7.4 and 7.3 months, respectively) compared to gemcitabine/nab-paclitaxel (5.7 months). OS was also higher for Nalirifox (11.1 months) compared to gemcitabine/nab-paclitaxel (10.4 months), although not significantly different from that of FOLFIRINOX [62].

**Table 3:** Comparative overview of chemotherapy regimens in metastatic PDAC.

Treatment Regimen	Median PFS (Months)	Median OS (Months)	Highlights/Notes
**FIRGEM (FOLFIRI.3 + Gemcitabine)** [60]	6.0	Not specified	Improved PFS vs. gemcitabine; higher hematologic toxicity
**FOLFIRINOX** [54]	6.4	11.1	Strong OS and PFS benefit compared to gemcitabine
**Gemcitabine + Nab-Paclitaxel (MPACT)** [55]	Not specified	Up to 42.0	Extended survival noted in long-term MPACT follow-up
**Gemcitabine + 5-FU/Erlotinib/Cisplatin/Capecitabine** [56,57,58,59]	Minimal to no benefit	Minimal to no benefit	No significant survival advantage over gemcitabine monotherapy
**Nalirifox (NAPOLI-3 trial)** [61]	Not specified	11.1 (vs. 9.2 for gem/nab-paclitaxel)	Superior to Gem/NabP; GI toxicity more common, less hematologic toxicity
**Meta-analysis by Nichetti et al.** [62]	Nalirifox: 7.4; FOLFIRINOX: 7.3; Gem/NabP: 5.7	Nalirifox: 11.1; FOLFIRINOX: 11.7; Gem/NabP: 10.4	Nalirifox and FOLFIRINOX show better PFS and OS than Gem/NabP
**Olaparib (BRCA-mutated patients, POLO trial)** [63]	7.4 (vs. 3.8 for placebo)	No OS improvement reported	First targeted maintenance therapy approved for BRCA-mutated metastatic PDAC patients

#### 2.4.5. Maintenance Therapy

A small but clinically meaningful subset—approximately 5% to 9%—of pancreatic cancer patients harbor germline or somatic mutations in *BRCA1* or *BRCA2*, which impair DNA repair via homologous recombination. These mutations confer therapeutic vulnerability to poly (ADP-ribose) polymerase (PARP) inhibitors. The phase III POLO trial evaluated Olaparib (PARP inhibitor) as a maintenance therapy in germline BRCA-mutated metastatic PDAC following first-line platinum-based chemotherapy. Olaparib significantly prolonged progression-free survival (7.4 vs. 3.8 months; HR = 0.53; *p* = 0.004), although no overall survival benefit was observed at interim analysis [7,63]. Despite this limitation, the findings were practice-changing. In December 2019, the U.S. FDA approved maintenance Olaparib for this specific population, marking it as the first biomarker-driven targeted therapy approved for pancreatic cancer [31]. This milestone highlights the growing potential of personalized therapy in a malignancy long regarded as therapeutically resistant.

## 3. Future Directions in Pancreatic Cancer: Therapies, Biomarkers, and Molecular Tools

### 3.1. The Dual Role of Galectin-1 in Pancreatic Cancer Microenvironment

As research into the tumor microenvironment advances, novel stromal targets are being explored. Recent preclinical studies have identified a novel role for Galectin-1 in pancreatic cancer, acting not only as an extracellular signaling molecule but also as a nuclear regulator that sustains KRAS expression in cancer-associated fibroblasts [64]. While promising, these findings are preliminary and require validation in larger studies before clinical translation.

### 3.2. Circulating Tumor DNA (ctDNA) in Pancreatic Cancer Management

Circulating tumor DNA (ctDNA) has emerged as a minimally invasive biomarker with significant prognostic value in PDAC. Detectable ctDNA is associated with shorter overall survival, minimal residual disease, and earlier recurrence [65]. Among the most frequently detected alterations in ctDNA are KRAS mutations, particularly in codons 12 and 13 (e.g., G12D, G12V), which occur in over 90% of PDAC cases [7,8]. Quantitative changes in ctDNA levels and variant allele frequency (VAF) can also reflect treatment response or disease progression [66]. A 2019 study involving 112 patients confirmed its utility in guiding adjuvant therapy decisions when assessed pre- or postoperatively [66]. The *AGITG DYNAMIC*-Pancreas trial further demonstrated the feasibility of using tumor-informed ctDNA to guide adjuvant chemotherapy after resection, identifying five weeks post-surgery as an optimal sampling time. ctDNA detection correlated with early recurrence, independent of traditional prognostic markers [67].

### 3.3. Targeted Therapies

KRAS mutations, particularly the G12D subtype, occur in over 90% of PDAC cases and have long been considered undruggable. However, recent advances have led to the development of KRAS G12D-specific inhibitors, such as MRTX1133 and RMC-9805. MRTX1133 is a selective non-covalent inhibitor that binds the inactive GDP-bound form of KRAS G12D and has demonstrated potent tumor regression in PDAC mouse xenograft models, including near-complete responses in some cases [8]. Similarly, RMC-9805 is part of a new class of RAS(ON) inhibitors that selectively target the active GTP-bound form of KRAS G12D. Preclinical studies showed strong anti-proliferative activity and tumor growth inhibition in PDAC models, and the compound recently entered a phase 1 clinical trial (NCT06040541) [8]. These agents represent promising tools in the precision oncology pipeline for PDAC and are being evaluated for safety and early efficacy in humans.

In addition to direct KRAS targeting, efforts to inhibit upstream and downstream effectors of KRAS are being explored. Upstream, inhibitors of SHP2 (e.g., TNO155, RMC-4630) and SOS1 (e.g., BI-3406) aim to block KRAS activation by disrupting interactions with guanine nucleotide exchange factors. Downstream, multiple agents are targeting the RAF-MEK-ERK and PI3K-AKT-mTOR pathways, which are hyperactivated in KRAS-mutant PDAC. For example, trametinib (MEK inhibitor) and everolimus (mTOR inhibitor) have shown modest preclinical activity, but their clinical efficacy has been limited by adaptive resistance and toxicity. Combination strategies involving these inhibitors alongside chemotherapy or immunotherapy are under active investigation to overcome pathway redundancy and feedback reactivation [8,68].

Tyrosine kinase inhibitors (TKIs) have been explored as well. The EGFR inhibitor erlotinib, in combination with gemcitabine, demonstrated a statistically significant but clinically modest improvement in overall survival (6.24 vs. 5.91 months; *p* = 0.038) in a phase III trial [58]. Recently, nimotuzumab, a humanized anti-EGFR monoclonal antibody, has shown immunologic activity in preclinical models, although its clinical efficacy in PDAC is yet to be proven [7]. Non-receptor tyrosine kinase inhibitors, including focal adhesion kinase (FAK) inhibitors and Bruton’s tyrosine kinase (BTK) inhibitors (e.g., acalabrutinib), are also being evaluated, primarily in combination with immune checkpoint inhibitors [7,8]. Acalaburtinib is under investigation in early-phase trials with pembrolizumab and chemotherapy, but data are still emerging, and the current findings are largely limited to early-phase trials and preclinical studies [9]. Additionally, a phase II study evaluated the efficacy of zolbetuximab, a monoclonal antibody targeting Claudin 18.2, a tight junction protein frequently overexpressed in PDAC [68].

In a subset of KRAS wild-type PDAC patients, rare gene fusion events represent actionable targets for precision therapy. RET fusions have shown sensitivity to selpercatinib and pralsetinib, both of which are selective RET inhibitors approved in other cancers and are currently under investigation in pancreatic cancer [69,70]. Similarly, NRG1 fusions, which activate HER3/HER2 signaling, are emerging as a particularly promising biomarker. Preclinical and translational studies have highlighted the oncogenic role of NRG1 fusions and their sensitivity to HER3-directed therapies [71]. The eNRGy trial, a phase II study of Zenocutuzumab—a bispecific HER2/HER3 antibody—demonstrated a 38% response rate in NRG1 fusion-positive PDAC, with a median PFS period of 5.6 months and overall survival of 12.4 months [72]. These findings underscore the importance of molecular profiling in identifying NRG1 fusions and integrating HER3-targeted therapies into treatment strategies for this rare but actionable subset.

Patients with germline BRCA1/2 or PALB2 mutations benefit from PARP inhibitors. Olaparib has shown improved progression-free survival as a maintenance therapy and is FDA-approved for BRCA-mutated PDAC [63]. Rucaparib is also recommended by the NCCN for patients with BRCA or PALB2 mutations in the metastatic setting [8]. Synthetic lethality is a promising approach to treating BRCA1/2 mutations, especially if sensitive to PARP inhibitors. These agents exploit deficiencies in homologous recombination repair, leading to the accumulation of DNA damage—specifically, double-strand breaks—by blocking the repair of single-strand breaks, ultimately resulting in tumor cell death [73].

The MTAP/PRMT5/CDKN2A axis represents another novel therapeutic avenue. Loss of MTAP and CDKN2A is common in PDAC, and PRMT5 inhibitors are being evaluated for their potential to exploit this vulnerability [6,7,68].

### 3.4. Immunotherapy

PDAC is traditionally viewed as non-immunogenic due to its dense stroma and immune-suppressive microenvironment. While immune checkpoint inhibitors (ICIs) have shown limited efficacy as monotherapies in PDAC, responses have been observed in 1–2% of cases with microsatellite instability-high (MSI-high) or mismatch repair-deficient (dMMR) tumors [6,7]. Recent work by Peshin et al. on immunotherapy in gastrointestinal malignancies underscores the clinical utility of PD-1/PD-L1 checkpoint inhibitors in MSI-H and PD-L1-positive tumors, reinforcing their potential relevance in biomarker-selected pancreatic cancer populations [74]. Agents such as pembrolizumab and nivolumab (anti-PD-1), as well as atezolizumab, durvalumab, and avelumab (anti-PD-L1), are under evaluation for their potential role in PDAC treatment [6,7,68]. A study by the Washington University School of Medicine evaluated BMS-813160, a dual CCR2/CCR5 antagonist, in combination with nivolumab, gemcitabine, and nab-paclitaxel for borderline resectable and locally advanced PDAC. BMS-813160 aims to inhibit tumor cell migration, proliferation, and invasion of tissue by blocking signal transduction pathways, enhancing the anti-tumor efficacy of combined immunotherapy [73]. A phase II randomized trial (1801 Part 3B) tested a drug called elraglusib (GSK-3β inhibitor) along with the standard chemotherapy combination of gemcitabine and nab-paclitaxel (GnP), comparing it to GnP alone. The results were promising, where patients who received elraglusib survived for a median of 10.1 months, compared to those on standard therapy, who survived for 7.2 months (HR 0.63, *p* = 0.01). While progression-free survival did not show a major difference, the 12-month survival rate was twice as high in the elraglusib group. Overall, elraglusib shows strong potential as a new first-line option in treating PDAC, although larger trials are required to confirm these findings [75]. A selection of ongoing clinical trials evaluating investigational therapies in PDAC is summarized in Table 4.

CTLA-4 inhibitors, including ipilimumab and tremelimumab, work by enhancing T-cell priming and activation. Their use in PDAC is being explored primarily in combination regimens, as monotherapy results have been modest [76,77,78]. Combination strategies involving ICIs with chemotherapy, SBRT, or CD40 agonists are under active investigation to overcome the immune-resistant microenvironment and improve clinical outcomes [6,68].

### 3.5. Adoptive Cell Therapies

Adoptive cell therapy (ACT), including chimeric antigen receptor T (CAR-T) cells and T-cell receptor-engineered T (TCR-T) cells, is emerging as a promising strategy in PDAC treatment [6,7]. CAR-T therapies target tumor-associated antigens including KRAS G12D, mesothelin, EGFR, HER2, and CLDN18.2, with early trials reporting partial responses and stable disease. However, challenges persist, including poor T-cell infiltration, the limited persistence of CAR-T cells, and risks of off-tumor toxicity. TCR-T cells, which recognize intracellular antigens presented on MHC molecules, have shown encouraging results [6,68]. A case report demonstrated a 72% reduction in the metastatic burden in a patient treated with KRAS G12D-targeted TCR-T cells [79]. Further optimization of these approaches is required to overcome PDAC’s immunological barriers.

### 3.6. Cancer Vaccines

Therapeutic cancer vaccines aim to stimulate tumor-specific immune responses and are being explored in PDAC as whole-cell (e.g., GVAX) [80], peptide-based (e.g., GV1001, MUC-1) [81,82], and dendritic cell-based vaccines [83]. The GVAX vaccine functions by secreting a granulocyte–macrophage colony-stimulating factor that promotes the infiltration of immune cells, particularly CD8+ and CD137+ cells, into the tumor microenvironment. GVAX is being evaluated in combination with urelumab (an anti-CD137 agonist) and nivolumab in PDAC cases. A phase I clinical trial at the University of Pennsylvania is evaluating the immune response to an autologous dendritic cell-based vaccine in patients with resected PDAC [68,73]. Although they show immunological activity, clinical benefits have been limited. Often combined with ICIs or chemotherapy to enhance their efficacy, cancer vaccines remain investigational but hold promising results as one of the multimodal treatment strategies [6,84].

### 3.7. mRNA Vaccines

Advances in mRNA vaccine technology offer new potential for the treatment of PDAC, a cancer typically resistant to immunotherapy. The personalized mRNA vaccine autogene cevumeran encodes up to 20 patient-specific neoantigens from resected tumor tissue. In a phase I trial led by Dr. Vinod Balachandran at the Memorial Sloan Kettering Cancer Center, this vaccine, combined with atezolizumab and mFOLFIRINOX chemotherapy (PD-L1 inhibitors), elicited robust T-cell responses in 50% of participants, with responders showing prolonged recurrence-free survival, in some exceeding three years post-treatment [84]. These results highlight the promise that mRNA-based vaccines could reduce the relapse risk, and a global phase II trial is ongoing to assess efficacy and safety in a larger cohort [85,86,87,88]. Peptide-based and mRNA vaccines targeting KRAS mutations are under investigation. Notably, the ELI-002 peptide vaccine, currently being evaluated in the AMPLIFY-7P phase II trial, has demonstrated promising results, including ctDNA clearance and robust KRAS-specific T-cell responses. Additionally, mRNA-5671 is being studied in combination with pembrolizumab in a phase I trial for KRAS-mutant PDAC [68].

### 3.8. Oncolytic Virotherapy

Oncolytic viruses are engineered or naturally occurring viruses that selectively infect and kill tumor cells while stimulating systemic anti-tumor immunity. In PDAC, several candidates are under evaluation. Pelareorep (a reovirus) has shown a clinical benefit when combined with pembrolizumab and chemotherapy. Other promising agents include HF10, VCN-01, and genetically engineered herpes simplex and adenoviruses. The other therapies mentioned beforehand also have the potential to remodel the tumor microenvironment, enhancing the efficacy of concurrent immunotherapy [6,84].

### 3.9. Matrix-Depleting Strategies

The fibrotic stroma of PDAC poses a significant barrier to therapeutic delivery. Matrix-depleting strategies aim to improve drug penetration and immune cell access. PEGPH20 targets hyaluronic acid within the tumor microenvironment and has been evaluated in phase II/III trials, although the results have been mixed. Alternative approaches utilizing metformin or nanoparticle-based systems are also being explored [6,89]. In addition to enzymatic stroma remodeling, pharmacologic strategies targeting tumor-associated fibroblasts and focal adhesion signaling are under investigation. A study at the Cancer Center at Johns Hopkins University is evaluating defactinib, a FAK inhibitor, in combination with pembrolizumab as a neoadjuvant or adjuvant therapy in PDAC. This combination aims to reprogram the tumor microenvironment and enhance the anti-tumor activity of PD-1 blockade [73].

### 3.10. Biomarker-Guided and Investigational Approaches

Biomarkers are essential for the future of personalized medicine in PDAC. Myeloid-derived suppressor cells (MDSCs), arginine metabolism, and indole-3-acetic acid (IAA) have been proposed as predictive and prognostic indicators. The inhibition of protein arginine methyltransferase 5 (PRMT5) enhances the efficacy of gemcitabine against PDAC cells by impairing DNA repair and synthesis, thereby promoting apoptosis [73]. Leukemia inhibitory factor (LIF), a cytokine implicated in chemoresistance, is another target of interest. Additional experimental strategies are being explored to enhance drug delivery and immune activation in PDAC. These include endoscopic tumor ablation (e.g., radiofrequency or cryoablation) combined with immune checkpoint inhibitors, aiming to induce immunogenic cell death. Similarly, electroporation-based methods, such as irreversible electroporation (IRE), are being studied for their potential to enhance immune cell infiltration and synergize with PD-1/PD-L1 blockade. These approaches remain in the preclinical or early-phase clinical stages [8,79]. The integration of biomarkers and experimental delivery technologies into clinical trial designs may allow for better patient stratification and improved therapeutic efficacy [8,79,89].

**Table 4:** Summary of selected clinical trials of emergent therapies in pancreatic cancer.

Therapy Type	Drug/Intervention	Target/Mechanism	Phase	NCT ID
**Targeted Therapy**	Fruquintinib	TKI	Phase 2	NCT05257122 [90]
	Anlotinib		Phase 2	NCT04718701 [90]
	Penpulimab + Anlotinib		Phase 2	NCT06051851 [91]
	Sunitinib		Phase 2	NCT06390826 [92]
	MRTX1133	KRAS G12D	Preclinical/early	NCT05737706 [93]
	RMC-9805		Phase 1	NCT06040541 [94]
	PF-07934040	panKRAS	Phase 1	NCT06447662 [95]
	IMM-1-104	MEK inhibitor	Phase 1/2	NCT05585320 [96]
	IMM-6-415		Phase 1/2	NCT06208124 [97]
	Olaparib	PARP inhibitor	Phase 2	NCT04548752 [98]
	Rucaparib		Phase 2	NCT03140670 [99]
	Fluzoparib		Phase 3	NCT04300114 [100]
**Immunotherapy**	Nivolumab	PD-1	Phase 1	NCT02309177 [101]
	Pembrolizumab		Phase 2	NCT02704156 [101]
	Durvalumab	PD-L1	Phase 2	NCT02879318 [102]
	Atezolizumab		Phase 2	NCT03193190 [103]
	Ipilimumab	CTLA-4	Phase 1	NCT01473940 [76]
	Tremelimumab		Phase 1	NCT00556023 [77]
	Ipilimumab		Phase 2	NCT00836407 [78]
	Mitazalimab	CD40 agonist	Phase 2	NCT04888312 [104]
	APX005M		Phase 2	NCT05419479 [105]
**ACT**	CAR-T	CEA	Phase 1/2	NCT04037241 [106]
		Claudin 18.2	Phase 1	NCT04404595 [107]
	TCR-T	KRAS G12D	Phase 1	NCT03745326 [108]
		TP53	Phase 1	NCT05877599 [109]
		Mesothelin	Phase 1	NCT04809766 [110]
**Vaccine**	GVAX	T-cell response	Phase 2	NCT00240262 [80]
	DC-based (WT1)	WT1	Phase 1	UMIN000040063 [83]
	KRAS peptide	KRAS	Phase 1	NCT04117087 [81]
	SVN-2B peptide	Survivin 2B	Phase 2	UMIN000021416 [82]

## 4. Conclusions

Pancreatic ductal adenocarcinoma remains a highly lethal malignancy, although recent advances in surgical techniques, chemotherapy, and molecularly targeted therapies have improved outcomes. Emerging immunotherapies and personalized treatment strategies based on molecular profiling offer additional promise. Continued progress will depend on earlier detection, tailored therapeutic approaches, and robust clinical trial participation. While challenges persist, the evolving therapeutic armamentarium provides cautious optimism for improved survival.

## Figures and Tables

**Table 1:** Summarizes the current classification of pancreatic cancer resectability and outlines the corresponding surgical management strategies. Importantly, locally advanced disease is distinct from metastatic disease and such patients may become surgical candidates following neoadjuvant therapy.

Resectability Status	Definition	Recommended Management
Resectable	No arterial involvement; ≤180° venous contact without contour irregularity	Upfront surgery followed by adjuvant therapy
Borderline Resectable	Limited venous or arterial involvement, potentially reconstructible	Neoadjuvant therapy → restaging → surgery if feasible
Locally Advanced	Encasement of major arteries or non-reconstructible veins	Non-surgical; systemic therapy ± radiation, potential downstaging
